# The value chain of the edible caterpillar *Elaphrodes lactea* Gaede (Lepidoptera: Notodontidae) in the Miombo forest of the Democratic Republic of the Congo

**DOI:** 10.1186/s13002-019-0319-y

**Published:** 2019-08-14

**Authors:** Olivier Bomolo, Saliou Niassy, Chrysantus M. Tanga, Auguste Chocha, Laetitia Tartibu, Mylor N. Shutcha, Baboy Longanza, Sunday Ekesi, David M. Bugeme

**Affiliations:** 1 Université de Lubumbashi (UNILU), Faculté des Sciences Agronomiques, Lubumbashi, Democratic Republic of the Congo; 2 International Centre of Insect Physiology and Ecology (ICIPE)-African Insect Science for Food and Health, Nairobi, Kenya; 3 Université Nouveaux Horizons (UNH), Lubumbashi, Democratic Republic of the Congo; 4 University of Johannesburg, Doornfontein Campus, Cnr Joe Slovo Drive and Beit St, Doornfontein, Johannesburg, Gauteng 2001 South Africa

**Keywords:** Entomophagy, Habitat loss, Conservation, Indigenous communities, Bemba, Non-timber product

## Abstract

**Background:**

*Elaphrodes lactea* Gaede is a highly praised edible lepidopteran insect in the Miombo forest in the DRC. Both caterpillars and pupae of this species are consumed. Following recent declines in the Miombo forest, it is crucial to investigate the rate of consumption, biological, and exploitation cycles, as well as the trade and profitability of *E. lactea* to develop a sustainable program for its use.

**Methods:**

We, therefore, embarked on a survey in 10 sites located in Lubumbashi between 2011 and 2015. Information on *E. lactea* supply chain and harvesting period was also documented as well as the mode of selling, pricing, and other determinants of the business. Data were analyzed using R2.15.0 software and means were compared using the Fisher LSD test.

**Results:**

The study revealed that *E. lactea* is the most preferred caterpillar and several indicators guide its exploitation. Caterpillars are available between March and April, and pupation starts in May. Harvesting starts within the household surroundings before reaching the bush, and several harvesting techniques are used. The indirect mode of trade of *E. lactea* is the most commonly used, with the average price/kg varying between USD2.32 (during in-season = production period for caterpillars) and USD5.24 (during dry season = off-season, mainly pupae). During the peak season of caterpillar production, the harvester’s average income per day varies between USD1.6 and USD3.0 whereas it varies between USD2.2 and USD5.2 during the pupal season.

Anthropogenic activities, coupled with climatic factors, constitute the main drivers affecting the availability of *E. lactea*.

**Conclusions:**

The study, therefore, calls on a concerted action from all stakeholders to increase awareness and the development of innovative measures for sustainable exploitation of this insect while ensuring rehabilitation of the forest through community participation.

## Introduction

Eating insects is a common practice in Africa. Insect consumption is considered as a potential means to solve food security on the continent. Globally, over 2000 insect species are reported edible; Africa alone has more than 500 edible insects [1].

The Democratic Republic of the Congo (DRC) is considered one of the most important hotspots of entomophagy in Africa and the world. Over 200 species of insects are reported edible in the country, including caterpillars, beetles, crickets, and etc. [1]. *Elaphrodes lactea* Gaede (Lepidoptera: Notodontidae), commonly referred to as “tunkubiu” in some of the local languages, is one of those edible caterpillars found in the province of Haut-Katanga. The species is univoltine, hence seasonal (caterpillars available between March and June) [2]. Although endemic to the Miombo forest, caterpillars thrive exclusively on leguminous trees, particularly *Brachystegia bohemii* Taub., *Julbernadia paniculate* Benth, *Isoberlinia angolensis* Benth., and *Albizia ferruginea* (Guill. and Perr.) Benth. [3, 4]. However, recent research has shown that *E. lactea*’s host range includes 19 plant species, of which some can also be found in the Bas-Congo Province (actually Central Congo) [5], Congo-Brazzaville [6, 7].

*Elaphrodes lactea* is not the only edible caterpillar in the region; nearly 28 species of caterpillars including *Lobobunaea saturnus* Fabricius and *Cinabra hyperbius* Westwood are also commonly consumed in DRC [5, 7]. Although *E. lactea* seems to be preferred because of its taste, its rate of consumption has never been compared with other available caterpillars [6] but the biology and life cycle of *E. lactea* have been studied in detail, including its nutritional value. Its lifecycle comprises five instars [6, 8]. One particularty of E. lactea is that both caterpilar and pupae are consumed.

The dearth of information on *E. lactea* supply chain, from collection to selling points in the markets, is alarming, yet recent reports on a decline in forest cover and deforestation suggest the potential scarcity and depletion of the caterpillar as a result of habitat change and overexploitation [7–9]. Several reports have shown that the species depends heavily on available host plant biomass; rearing experiments showed that monthly leaf consumption by *E. lactea* was 733 m^2^/ha (dry weight of 98 kg/ha) and the dry weight of their feces was 90 kg/ha. Hence, habitat loss might not only prevent the insect from regenerating, but also expose both adults, caterpillars, and pupae to ecological relations that may affect its availability in the longer run [10].

Understanding the ecological importance of edible caterpillars is a critical step towards the preservation of biological diversity. More importantly, the contribution of *E. lactea* to food security, in the context of rising demand for protein in the country, is of paramount necessity. Considering the apparent high preference for this insect (both caterpillars and pupae are consumed), it seemed desirable to investigate the exploitation and trade, in order to develop a sustainable approach for the exploitation of *E. lactea*.

The outcome of such an investigation will undoubtedly raise awareness among the various stakeholders and players involved along the supply chain of *E. lactea*, serving as an impetus to engage the community in ecosystem preservation, habitat management through agroforestry, and income generation through regulatory measures.

## Materials and method

### Study sites

**Fig. 1:**
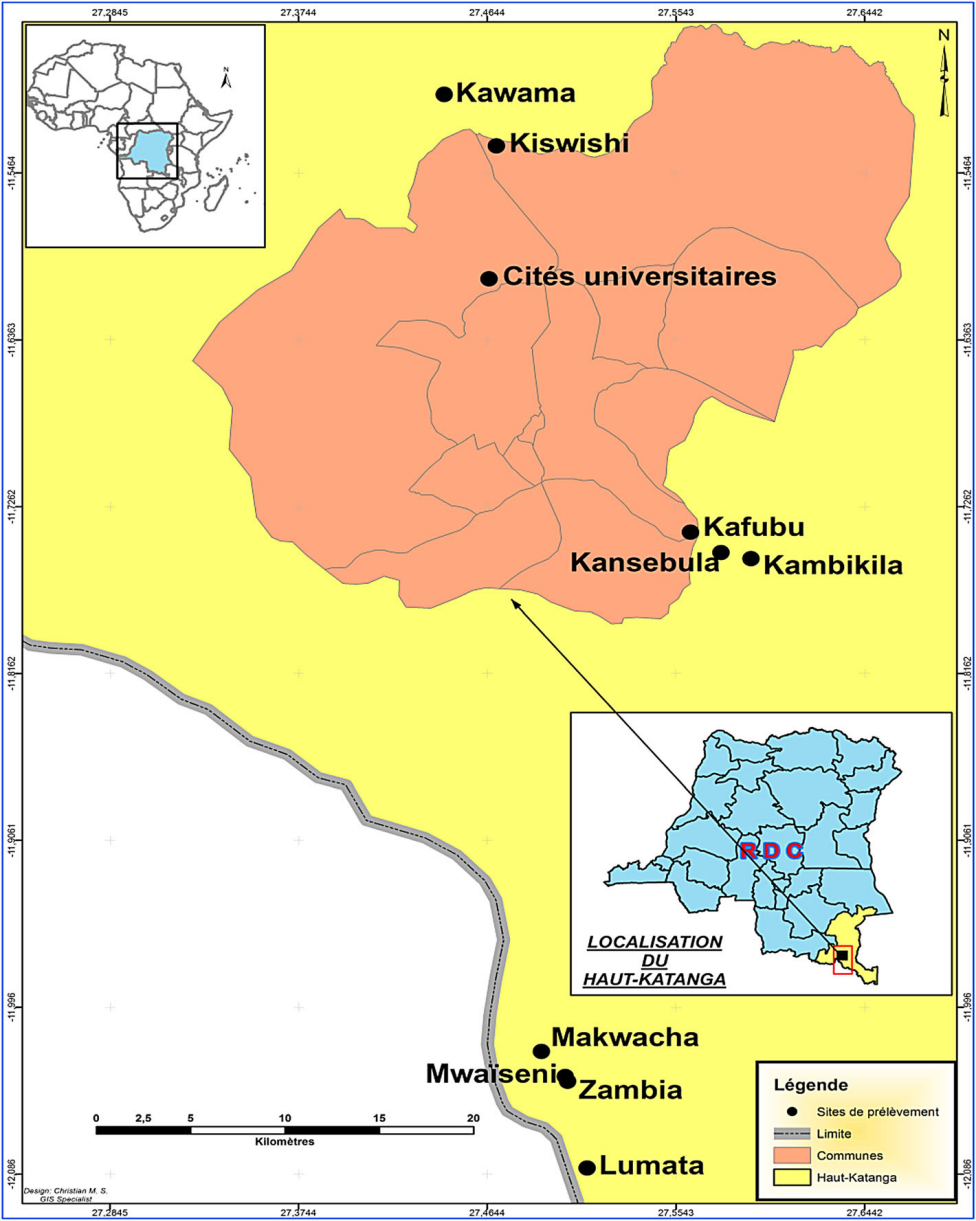
Study sites

The study was conducted in the Plain of Lubumbashi at the following sites: Kawama, Kiswishi, University Campus, Mwaiseni, Kafubu, Kansebula, Kambikila, Makwasha, Zambia, and Lumata (Fig. 1). These sites are located within the Miombo ecosystem, and they are also considered major collection centers of edible insects, supplying Lubumbashi and the eastern part of the country. The university campus was selected considering its status as a dedicated *E. lactea* research stations between 1960 and 1980. The minimum and maximum distances between the center of the city of Lubumbashi and these sites are 7 km and 45 km.

### Vegetation, climate, and soil

**Fig. 2:**
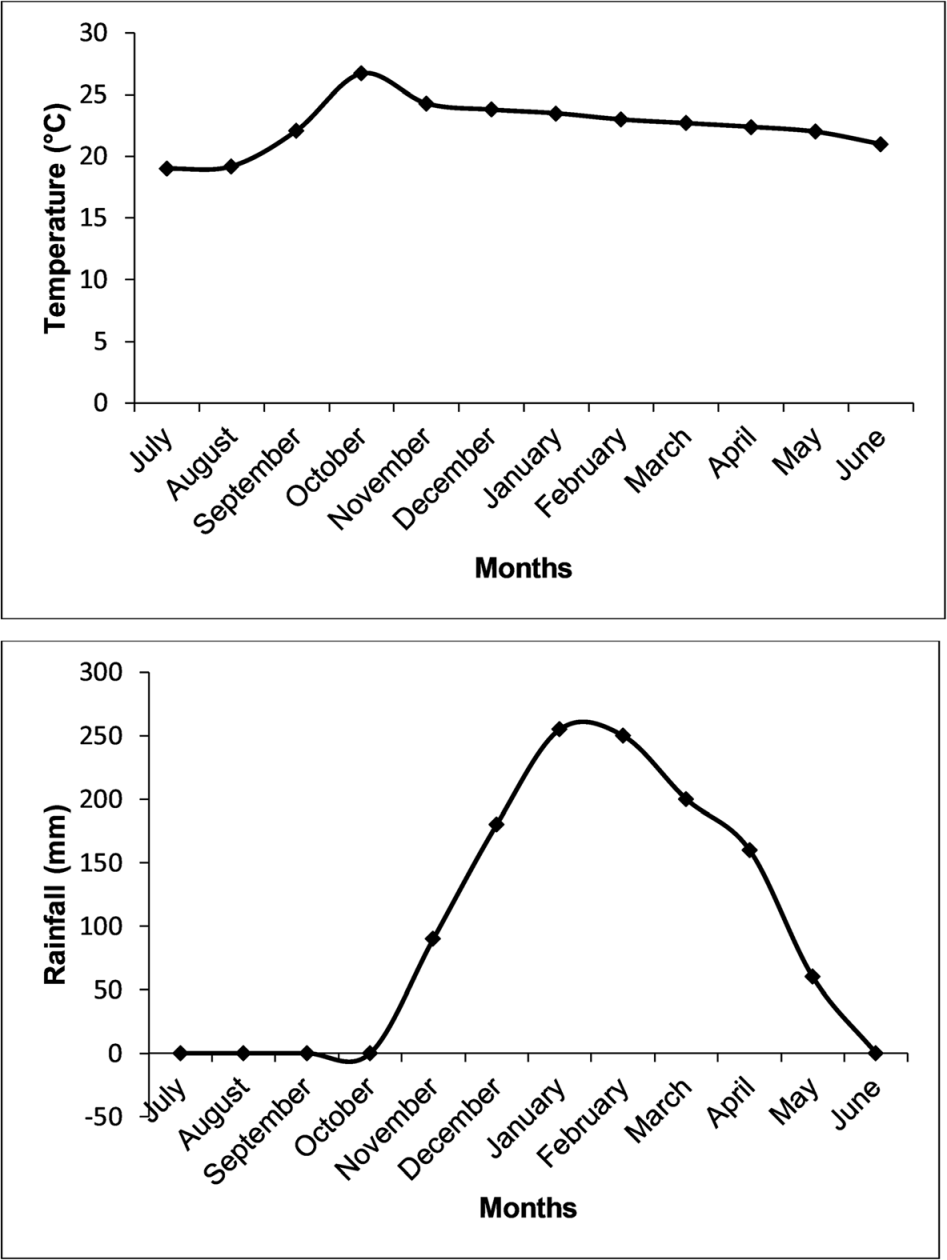
Monthly mean temperatures (°C) and monthly average rainfall (mm) in the city of Lubumbashi (Haut-Katanga) 2012–2017

The climate is subtropical, characterized by heavy rainfall from November to April and dry conditions from May to September. The city receives an annual average rainfall of approximately 1300 mm with a maximum of 1200 mm during the rainy season. The average annual temperature is about 20 °C. The lowest daily temperature is 15 °C at the beginning of the dry season. The monthly mean temperatures and monthly average rainfall (mm) in the city of Lubumbashi between 2012 and 2017 are presented in Fig. 2.

The period between September and November is warm (September and October are the hottest months with temperatures ranging between 31 °C and 33 °C), and from May to August the weather is cool [11]. Daily temperatures vary considerably, especially during the cold season when night-time temperature readings drop to near freezing—as low as 5 °C [12]—and daytime temperatures of 24 °C are not uncommon. At the beginning of the dry season, the herbaceous vegetation dries up, except drought-resistant plant species and those growing in the wetlands with permanent pools of water.

### Major communities living in the area

Katanga is the country’s second most important economic city after Kinshasa. The population is composed of different ethnic groups such as Lamba, Sanga, Bemba, and Kaonde. These tribes subsisted by fishing, trade, mining, and agriculture. Mining represents the primary driver of the economy of this province. The population is relatively well educated compared with other provinces of the country, especially at the primary school level; however, secondary school is more challenging for young boys and girls. The employment rate for women is 62.8% compared with 60.2% for men, while respective average monthly incomes amount to US dollars 19.0 and US dollars 32.0 for men [13].

### The survey

In order to understand the various factors governing the trade of *E. lactea* caterpillar, 900 individuals were interviewed between 2011 and 2015, using a questionnaire structured in the form of a guide. The interviewees were selected on the basis of the regular distribution method described by Dagnelie [14]. The age bracket of participants was between 18 and 50, and the gender ratio between men and women was 3:1.

### The questionnaire

The questionnaire was designed to capture information such as main indicators of harvesting season, collection sites, methods of harvesting, economic drivers, profit, pricing, and parallel activities. Information on preference in the consumption of *E. lactea* was compared with information recorded on other caterpillars also consumed in the region. Information on the most suitable season for harvesting and collecting of caterpillars and pupae, as well as the study site and its distance from the central district and principal city of Lubumbashi, was also captured. Data on vegetation (abundance of *B. bohemii* (Bb), *J. paniculata* (Jp), and other host plants per ha (%), as well as their diversity and seasonal occurrence according to the various stages of the *E. lactea* lifecycle, was recorded. In order to determine the value of *E. lactea* harvesting in relation to other forest activities, attributes such as primary or secondary harvesters, the origin of cultural information were captured (cultural heritage).

The study also investigated the different methods of harvesting used by the collectors of both caterpillars and pupae, as well as information on the selection of the harvesting sites and the understanding of the spatial ecology of the caterpillar.

The survey looked at financial drivers that determine the consumption of *E. lactea*, for example, quality (freshness, color. stage), buyer attributes, and quantity sold per day. Information on the supply chains (or mode of trade) was covered in the questionnaires, including the number of trips, the most frequented sites, and the frequency of visits. The number of visits (max/min) per site, distance traveled by the gatherers and means of transport, average collection time and the main family member involved in this activity (gender, adult, or teenager) were examined along with the possibility of camping on site. Furthermore, information on the measuring containers (size, volume) and the related selling price was captured.

Information on the cost of living and the net income of the fresh harvest and alternative costs, as well as data on the duration of the harvesting period, were also documented along with the mode of selling (wholesale or retail). In order to determine the cost-benefit analysis, the quantity of *E. lactea*, surface area, number of host plants, quantity harvested per day, the proportional surface area required, and trees were investigated. The price/container and the trends in the month and year were also included.

In order to correlate the exploitation of *E. lactea* and the production of firewood, the diversity, and quality of trees used as firewood, as well as the trend of firewood production in relation to *E. lactea* production over the past 10 years, were investigated. The questionnaire also looked at the willingness to replant trees and the awareness of the magnitude of the exploitation of forest resources among respondents. The particulars of all participants in the survey were captured for further investigation.

### Statistical analyses

The statistical analyses were performed using R2.15.0 software. Harvesting and commercialization (trade, income, revenue per day) parameters were analyzed using ANOVA. Means were compared using the Fisher LSD test (multiple post hoc) at *p* = 0.05. In this study, 1 US Dollar equals to 1500–1700 Congolese Franc [15].

## Results

### Preference and frequency of consumption of caterpillars in Haut-Katanga

**Fig. 3:**
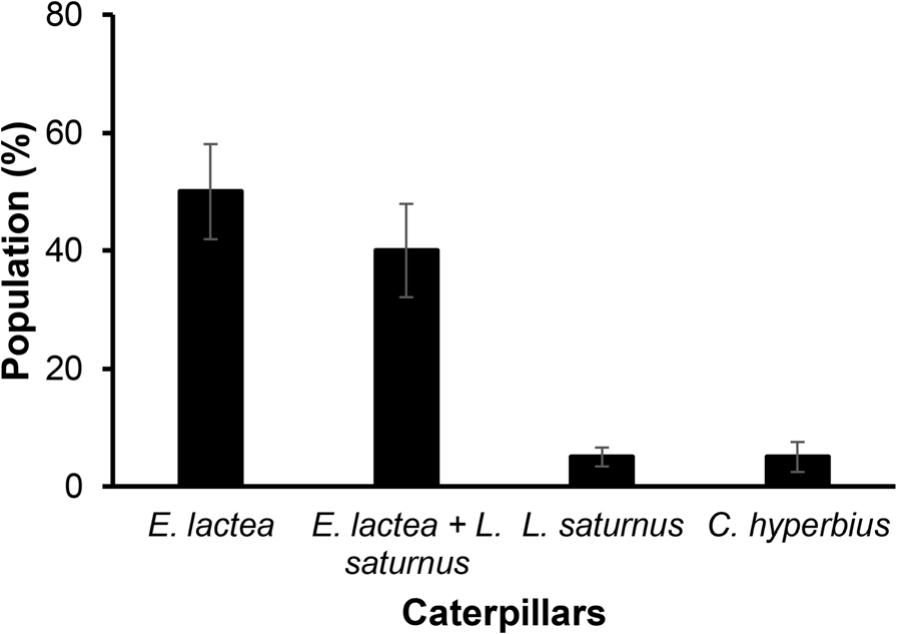
Preference in the consumption (±standard error) of caterpillars in the city of Lubumbashi, (consumption mean at least 500 g/day)

Of the three main species of caterpillars consumed in Haut-Katanga, *E. lactea* is the most popular. The other species, namely, *L. saturnus* and *C. hyperbius*, are less frequently used as food; however, a mixture of *E. lactea* and *L. saturnus* is typical (40%) (Fig. 3).

**Fig. 4.**
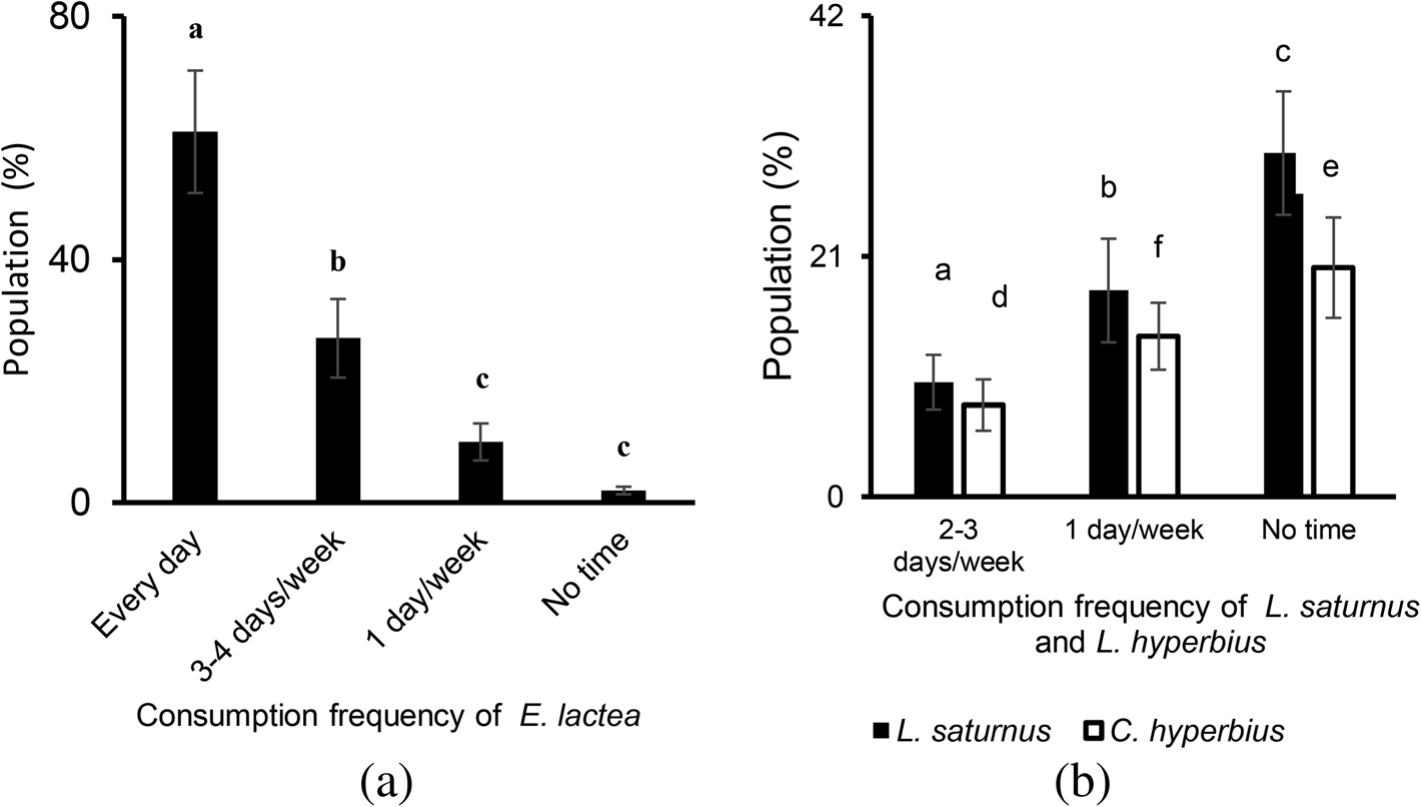
Frequency of consumption (±standard error) of caterpillars (*E. lactea* (**a**); *L. saturnus* and *C. hyperbius* (**b**)) by the population of the city of Lubumbashi (consumption mean at least 500 g/day)

The majority of respondents (61%) eat *E. lactea* every day while 27% consume it 3 to 4 days per week (Fig. 4a).

Concerning *L. saturnus* and *C. hyperbius*, only 10% and 8% of the population respectively consume them two to three times per week. The majority of respondents reported limited or no consumption at all of the other two caterpillar species (48% and 34% respectively) (Fig. 4b).

### Indicators of availability and seasonal cycle of exploitation of *E. lactea* in Haut-Katanga

The main indicators for the presence of *E. lactea* are presented in Table 1. Leaf damage and direct communication are the main drivers indicating the onset of the harvesting of caterpillars in the various sites surveyed. However, in Kafubu and Kansebula, in addition to the criteria mentioned above, humidity and abundance of host plants seem to play an essential role as indicators for caterpillar harvesting.

**Table 1:** Indicators of the harvesting season and factors perceived to affect the production of the *E. lactea* caterpillar in the city of Lubumbashi

	Indicators of the harvesting season				Factors perceived to affect the production			
Indicators	Leaves Consumed by *E. lactea*	Strong Humidity	Strong Abundance of Food Plants	Information verbal communication	Climate	Intensity of Harvesting	Intensity of Collection	Anthropogenic Activities
Sites								
University Campus	+			+	+	+	+	+
Kawama	+			+	+		+	+
Kafubu	+	+	+	+	+		+	+
Kambikila	+		+	+	+			+
Kansebula	+	+	+	+	+		+	+
Kiswishi	+	+		+	+			+
Makwasha	+	+		+				+
Mwaiseni	+			+	+		+	+
Lumata	+		+	+	+			+
Zambia	+	+		+	+	+	+	+
% Respondents	100	50	40	100	90	20	60	100

**Fig. 5.**
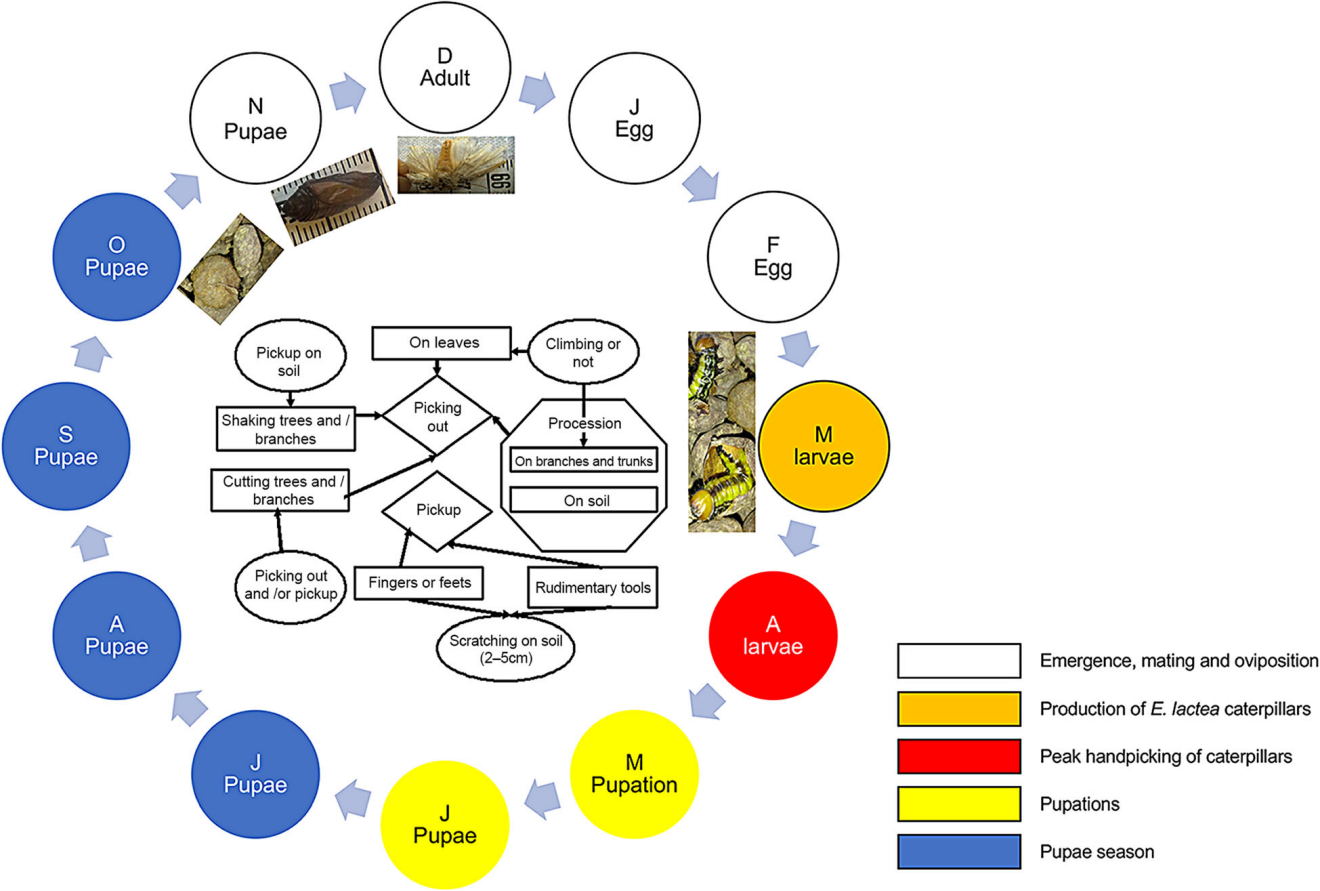
Diagram describing the annual availability and the harvesting of caterpillars and pupae of *E. lactea* in Haut-Katanga

The biological and exploitation cycles are presented in Fig. 5. According to the respondents, adults of *E. lactea* are found between December and January, and eggs are deposited around February on the leaves of suitable host plants. Newly hatched caterpillars are also found feeding on the leaves between March and April before entering a pupation phase between May and June. The pupae then diapause until November before the new emergence of adults is observed (Fig. 5).

### Harvesting and collection of *E. lactea* caterpillars and pupae

The production of *E. lactea* is heavily dependent on its lifecycle (seasonality). The methods of collection vary between March and April, and caterpillars can be collected from the leaves or the soil by handpicking, shaking off the trees and branches, or by cutting the trees or branches. In May–June, pupating caterpillars and pupae are collected from the soil by handpicking or with the help of rudimentary tools used to scratch the soil (2–5 cm deep). There is even one unusual technique that is used which involves the harvester scratching in the ground with his or her toes to locate the caterpillars, after which they are picked up (Fig. 5). Collecting pupae continues from July to October (Fig. 5).

### Business and trade models of *E. lactea*

**Fig. 6.**
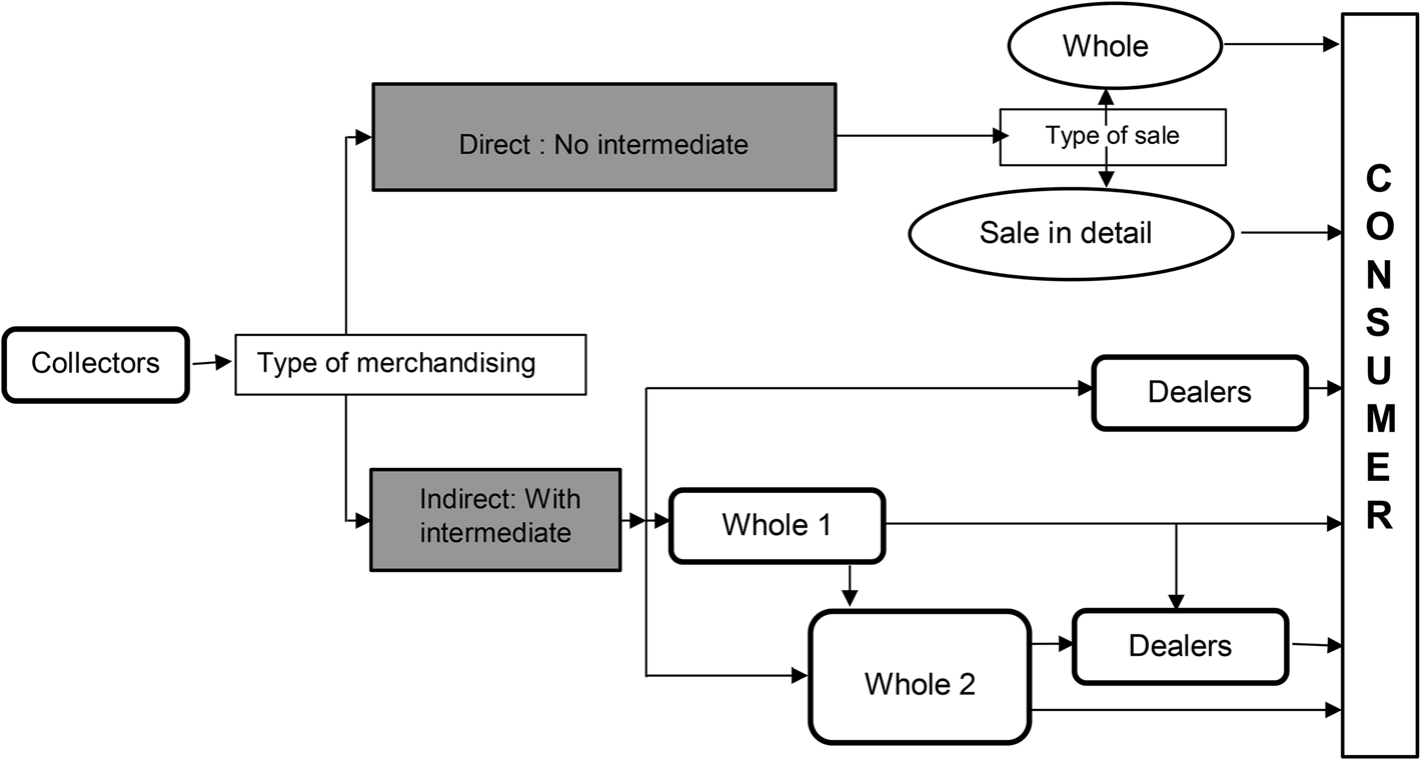
Diagram describing the merchandising circuit of *E. lactea* in Lubumbashi

At the beginning of the *E. lactea* season, the harvesting of larvae often takes place in areas surrounding the households, but as the season unfolds, collection sites move further into the thick forest. Figure 6 describes the various commercialization pathways of *E. lactea.*

**Fig. 7.**
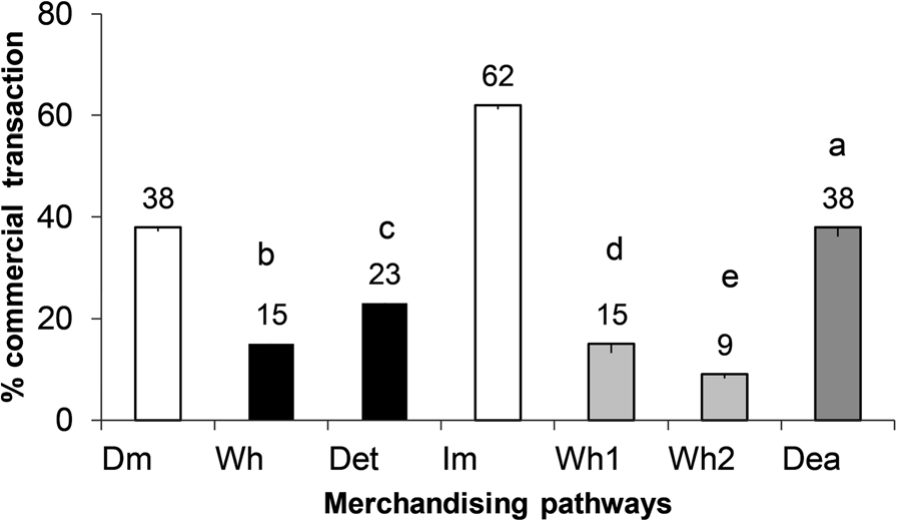
Chart showing the proportion of transaction according to the merchandizing pathways (Direct or indirect) of *E. lactea* in Lubumbashi

Indirect trade is the most common mode of trade, as confirmed by 62% of the people engaged. Of the indirect traders, 38% are dealers, 15% first-level wholesalers (whole 1) and 9% second-level wholesalers (whole 2) (Fig. 7).

A Fisher pairwise comparison test LSD showed a significant difference between the two models according to the population interviewed (df = 6; *p* < 0.05). In the direct business model (38%), 23% of transactions are made through retail selling whereas 15% of transactions take place through wholesale (Fig. 7).

### Monthly price variations of *E. lactea* and pupae/kg and daily revenue of harvesters in Haut-Katanga

The average prices of caterpillars on the markets of the city of Lubumbashi (Mze Laurent Désiré Kabila, Kenya, Market of Rail, Mimbulu Market, Zambia Market, Kipushi, and other markets in downtown Lubumbashi), are USD2.32/kg (during rainy season = period of production) and USD5.24/kg (during dry season = off-season).

**Fig. 8.**
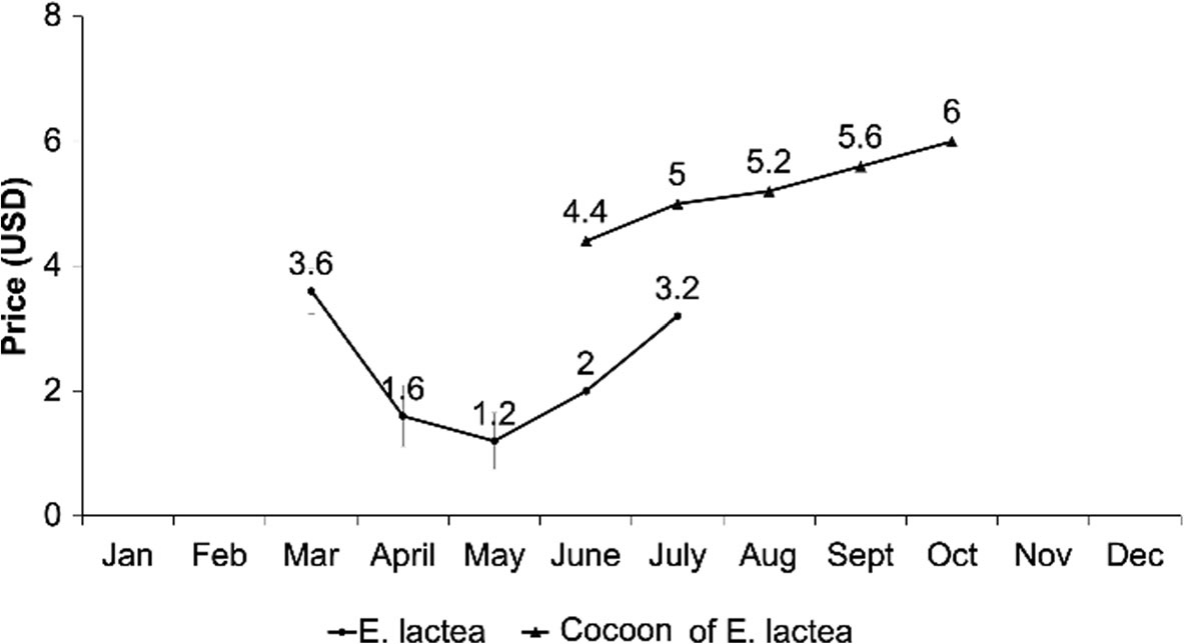
Monthly variations in price (US dollars) of *E. lactea* caterpillars and cocoons/kg on the markets of Lubumbashi

The lowest possible market prices of *E. lactea* per kilogram in-season and off-season are USD1.2/kg and USD4.4/kg. The highest possible market prices of *E. lactea* are USD3.6/kg (caterpillars: in-season) and USD6/kg (pupae: off-season) (Fig. 8).

Overall, the production of *E. lactea* in the off-season is more profitable than in the regular season of production (caterpillars: March–July). The mean comparison of the two seasons showed some significant differences (df = 9, *p* < 0.05).

**Fig. 9.**
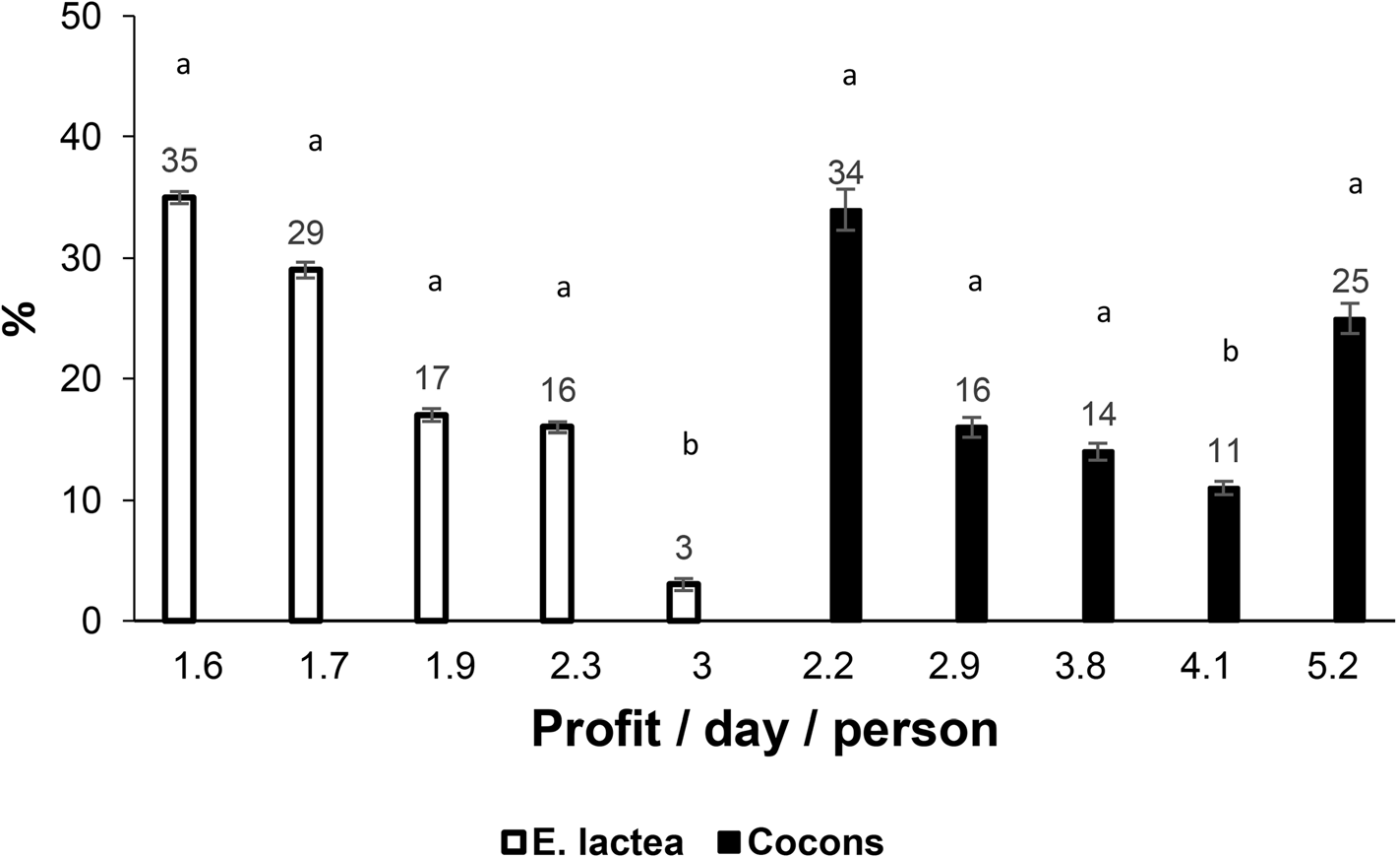
Revenue (US dollars) per day by those responsible for the collection of caterpillars and cocoons

During the pupae season, the financial gain varies between 37.5 and 73.3%, as the average income per day of harvesters varies between USD2.2 and USD5.2 (Fig. 9). During the peak season of production, the average income per day of harvesters varies between USD1.6 and USD3.0 (Fig. 9).

### Different categories of *E. lactea* harvesters in Lubumbashi

**Fig. 10.**
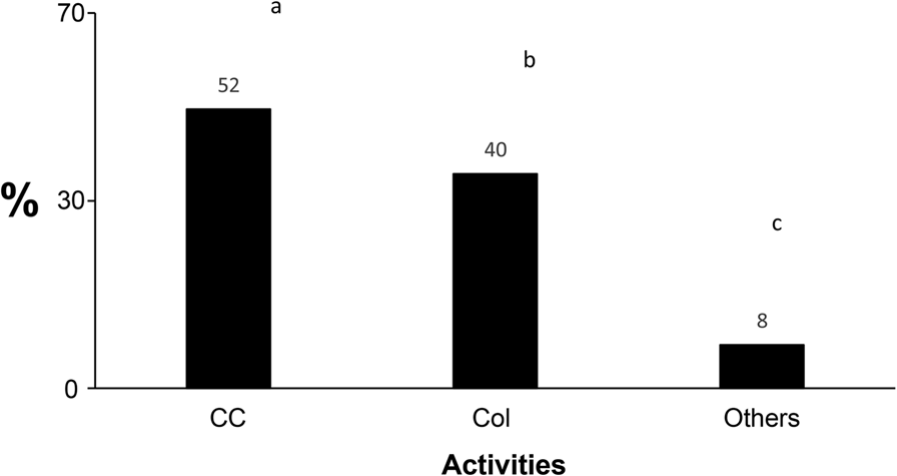
Different categories of harvesters of *E. lactea* in the DRC

There are three groups of harvesters of *E. lactea*, namely, subsistence harvesters (52.0%) committed to the collection of the insects for consumption and trade, gatherers (40.0%) consisting of villagers and indigenous people who also collect the insects for trade, and finally petty collectors, farmers, and hunters (8.0%) who occasionally harvest caterpillars in the forest. There is a significant difference in the level of harvesting between these different communities in the forest (df = 2, *p* < 0.05) (Fig. 10).

### Factors affecting the production of *E. lactea* in Haut-Katanga

In all study sites, except for Makwasha, it was noted that climatic conditions constitute the most significant factors affecting *E. lactea* production. However, anthropogenic activities were also pointed out as the main threat to the *E. lactea* population in Haut-Katanga (Table 1). It has been noted that intensive harvesting of caterpillars and pupae affect the availability of caterpillars in the subsequent seasons.

## Discussion

The consumption of caterpillars is a part of the country’s cultural heritage and has been practiced over many generations. The study showed that *E. lactea* is most preferred caterpillar compared with *L. saturnus* and *C. hyperbius* thereby confirming previous findings [7, 9, 16]. This study showed also that *E. lactea* pupae have a better taste, which accounts for the higher preference. Nutritional analysis of *E. lactea* by Malaisse in the 1970s revealed a lipid content as high as 29.6%, more than twice the values reported for most other caterpillar species. It is also a significant addition to the protein supply of rural people.

Pupae are more preferred than caterpillars. In that regard, Malaisse [16] demonstrated already that the absence of the digestive content in the pupae would give them a pleasant taste, giving the consumers an outstanding gustatory quality. Taste, texture, and visual appearance were all highlighted by Ghosh et al. [17] as important determinants of an edible insect’s acceptability as a food item for human consumers. Besides, consumers indicated that better appearance makes it less frightening compared with other caterpillars.

The indicators of the harvesting season of caterpillars in the Plain of Lubumbashi are mainly the presence of damaged leaves and the level of humidity in conjunction with the abundance of food plants (and also the bushy aspect of the canopies). Information on the presence of caterpillars is verbally disseminated across the region; hence, climate plays a vital role as an indicator of the *E. lactea* harvesting season, as indicated by Malaisse [18].

The *E. lactea* lifecycle is 12 months (from December to November). The period of incubation is about 1 month (from mid-January to mid-February), the larval stage is 4 months (from mid-February to mid-May), the pupal stage is 6 months (from mid-May to mid-November), while the adult stage is 2 months (mid-November to mid-January). This corroborates previous reports [18, 19], except that in this study, it was found that *E. lactea* life cycle shifted by 1 month earlier. It is suspected that the shift in the lifecycle might be due to recent changes in climatic conditions that are currently taking place in the Miombo ecosystem. These changes are mainly due to anthropogenic activities (mining, constructions, farming, overexploitation), which result in habitat changes that the insects are exposed to [20].

In this study, several factors were found to affect trade, including distance (to market or harvesting site), the potential value of the market, number of transactions, stage (taste), transport, and number of intermediaries (wholesalers). The harvesters and the collectors move to the sites on foot and use several techniques and tools for harvesting, including sticks, branches, and tablespoons.

The displacement from households towards the areas of harvest is achieved on foot or by bicycle. These two means of transportation help the harvesters go farther into the forest where vehicles cannot reach. Dikumbwa and Kisimba [20] demonstrated that the quantity of caterpillars harvested is a function of the transport means used. The longer the distance between the households and the points of harvest, the fewer the caterpillars. The latter fact differs from the quantity harvested close to households where the entire community, including men and children, are involved in the collection of caterpillars.

Besides the long-distance accomplished by harvesters, the methods of *E. lactea* harvesting are rudimentary. In most cases, harvesters use pieces of wood that they find in the sites of harvest, especially when harvesting takes place at long distances (between 20 and 45 km). Caterpillar harvesters are hardly ever keen to change collection sites. They are sedentary and rarely to move from one village to another, or from one site to another, even in the case of the disappearance of the caterpillar [20]. This explains, at times, the presence of caterpillars in some villages yet none in others. Production is not always quantified. It is rare that harvesters can provide an exact number in terms of kilograms of caterpillars harvested. This can be ascribed to the low level of education and lack of initiatives observed in these sites.

Pupae harvested in the off-season are highly praised, as mentioned above, due to their pleasant taste. For the case of *E. lactea*, the pupae are more preferred than the caterpillars. As a commodity, the higher the supply, the lower the price and vice versa.

Irrespective of the season, the income of caterpillar harvesters in the Plain of Lubumbashi is always lower than the minimum universal average wage, as per the indicators of human development [22].

The harvesting rates of caterpillars not only vary according to the types of players involved, but also the period of production. The month of March, with the presence of rains, brings the production of several other food commodities, including other edible insects, which put the caterpillars of *E. lactea* under immense competition with other foods. This also explains the variations of prices in the sites of production and consumption. Also, during the off-season, with a decrease in the production of many food commodities on the one hand and of the excellent taste of the *E. lactea* pupae on the other, the latter is sold at more elevated prices than during the period of full production of the larvae at the sites.

There is a range of harvesters, and this activity is not assigned to only a specific group or community. The first group of people harvest caterpillars while producing coal. The second group only collects caterpillars, while the third group harvests caterpillars only occasionally during their passages through the forest. The diversity of harvesters also implies heavy pressure on the availability of the insect.

The trade in *E. lactea* has in the past been interrupted due to the temporary disappearance of the insect in 2004 in the Plain of Lubumbashi. It reappeared in 2005. This is due to many factors: the region is generally populated by Bemba communities, native to the Plain of Lubumbashi, who are basically not farmers and therefore exploit the Miombo forest for primary needs. Therefore, the slashing of trees for charcoal production is a prevalent practice, and it is associated with caterpillar harvesting in the region, as reported by Dikumbwa and Kisimba [20]. Malaisse noted the dependency of this species on Miombo forest biomass and suggested forest protection policies. At the same time, he denounced the invasion of the *E. lactea* habitat by anthropogenic activities [10]. Concerns on Miombo forest decline have been raised in several countries sharing this biome and the potential decline of edible caterpillars [7, 8]. The forest indeed contributes to the livelihood of millions of people; therefore, there is a need to develop a strategic plan to sustain the ecosystem services [21]. Further research on the relationship between forest decline and the availability of *E. lactea* would be crucial for the future of this insect in the Katanga region. The research will use historical data on forest cover, remote sensing, and mapping to illustrate the magnitude of the decline to guide the decision.

## Conclusion

Lepidopterans are among the most prominent group of edible insects in Africa [23]. In Lubumbashi, *E. lactea* is one of the most preferred caterpillars, with both caterpillars and pupae being consumed and harvested throughout the year. Although perceived as an alternative solution to poverty in the Plain of Lubumbashi, the business of the *E. lactea* caterpillar is, however, not very lucrative. The exploitation of the caterpillar remains basic and involves much environmental degradation. The combined harvesting of various player and anthropogenic activities such as charcoal production puts the species at heavy risk. The value chain of *E. lactea* is similar to that of Mopane worm *Imbrasia belina* in Southern Africa and measures for sustainable management of this important insect have been studied and implemented in the region [24, 25]. This study, therefore, calls on a concerted action from all stakeholders to increase awareness and the development of sustainable *E. lactea* harvesting models through community participation to preserve the ecosystem and reduce the pressure on *the* forest.

## Data Availability

Please contact the author for data requests
